# Noisy Galvanic Vestibular Stimulation Combined With a Multisensory Balance Program in Older Adults With Moderate to High Fall Risk: Protocol for a Feasibility Study for a Randomized Controlled Trial

**DOI:** 10.2196/32085

**Published:** 2021-10-05

**Authors:** Ruth McLaren, Paul F Smith, Sue Lord, Preet Kamal Kaur, Yiwen Zheng, Denise Taylor

**Affiliations:** 1 Rehabilitation Innovation Centre School of Clinical Sciences Auckland University of Technology Auckland New Zealand; 2 Department of Pharmacology and Toxicology School of Biomedical Sciences University of Otago Dunedin New Zealand

**Keywords:** older adult, balance, rehabilitation, noisy galvanic vestibular stimulation, nGVS, brain stimulation

## Abstract

**Background:**

Reduced mobility and falls are common among older adults. Balance retraining programs are effective in reducing falls and in improving balance and mobility. Noisy galvanic vestibular stimulation is a low-level electrical stimulation used to reduce the threshold for the firing of vestibular neurons via a mechanism of stochastic resonance.

**Objective:**

This study aims to determine the feasibility of using noisy galvanic vestibular stimulation to augment a balance training program for older adults at risk of falls. We hypothesize that noisy galvanic vestibular stimulation will enhance the effects of balance retraining in older adults at risk of falls

**Methods:**

In this 3-armed randomized controlled trial, community dwelling older adults at risk of falling will be randomly assigned to a noisy galvanic vestibular stimulation plus balance program (noisy galvanic vestibular stimulation group), sham plus balance program (sham group), or a no treatment group (control). Participants will attend the exercise group twice a week for 8 weeks with assessment of balance and gait pretreatment, posttreatment, and at 3 months postintervention. Primary outcome measures include postural sway, measured by center of pressure velocity, area and root mean square, and gait parameters such as speed, step width, step variability, and double support time. Spatial memory will also be measured using the triangle completion task and the 4 Mountains Test.

**Results:**

Recruitment began in November 2020. Data collection and analysis are expected to be completed by December 2022.

**Conclusions:**

This study will evaluate the feasibility of using noisy galvanic vestibular stimulation alongside balance retraining in older adults at risk of falls and will inform the design of a fully powered randomized controlled trial.

**Trial Registration:**

New Zealand Clinical Trials Registry (ACTRN12620001172998); https://www.anzctr.org.au/Trial/Registration/TrialReview.aspx?id=379944

**International Registered Report Identifier (IRRID):**

DERR1-10.2196/32085

## Introduction

### Background

The contribution of age-related decline in postural responses to reduced mobility and falls in older adults is well established [1-4]. The mechanisms underlying this deterioration are complex but are thought to include impaired function and processing of sensory information from the visual, vestibular, and somatosensory systems. The vestibular system is perhaps the least understood of these balance systems. However, rich connections between the hippocampus, cerebellum, and brainstem suggest that treatments designed to enhance the vestibular system have the potential to improve postural stability and balance [5-8].

Noisy galvanic vestibular stimulation is a subsensory galvanic stimulation delivered with a superimposed Gaussian noise signal. The proposed mechanism of action is based on the principle of “stochastic resonance,” in which noisy galvanic vestibular stimulation enhances weak sensory input signals from the vestibular apparatus [9,10]. This then enhances vestibular perception and vestibulospinal reflex function, both essential components of the sensory feedback loop for balance [11-13].

All sensory systems decline with age [14]; however, there is strong evidence that balance training can reduce the risk of falls in older adults and improve clinical tests of stability [15-17]. Although there are known benefits of rehabilitation programs for balance retraining, less is known about the effect of noisy galvanic vestibular stimulation on postural control [18,19]. Emergent research in humans suggests immediate benefits to static balance. Inukai et al [20] reported immediate improvements in standing balance when noisy galvanic vestibular stimulation was administered to healthy older adults, and Iwasaki et al [4] demonstrated a similar positive effect in patients with bilateral vestibular dysfunction. There is some evidence that this benefit may be sustained. Fujimoto et al [21] examined the effect of 30 minutes of noisy galvanic vestibular stimulation on balance and found that a positive effect was maintained for up to 3 hours after exposure to the intervention. These improvements were further enhanced by a subsequent 30-minute exposure, which also sustained the beneficial effects. However, this sustained effect has not been replicated consistently in other studies; Inukai et al [22] found a poststimulation effect in contrast to Nooristani et al [23] and Keywan et al [24] who did not. A secondary analysis by Fujimoto et al [21] discovered that noisy galvanic vestibular stimulation resulted in significantly greater improvements in participants who were more unsteady on initial assessment [25]. This suggests that either noisy galvanic vestibular stimulation has a greater effect on the hypofunctional vestibular system or that different noisy galvanic vestibular stimulation parameters and experimental designs play a role in these findings. Noisy galvanic vestibular stimulation has also shown positive effects on walking balance, with Wuehr et al [12] and Iwasaki et al [26] finding that noisy galvanic vestibular stimulation resulted in an immediate positive effect on gait and in adults with bilateral vestibular pathology. However, to date, there are no studies of noisy galvanic vestibular stimulation in older adults who present with a fall risk, and no studies have investigated the repeated application of noisy galvanic vestibular stimulation as a clinical intervention. No study has investigated the augmented effect of concurrent noisy galvanic vestibular stimulation and balance rehabilitation to improve stability. We plan to redress these limitations by examining the impact of a balance rehabilitation program augmented with noisy galvanic vestibular stimulation in older adults at risk of falls.

### Objectives

This study aims to assess the feasibility of a future definitive randomized trial examining the effect of balance rehabilitation augmented with noisy galvanic vestibular stimulation in older adults at risk of falls. The primary objective is to assess the feasibility of the study process, including (1) rate of participant recruitment, conversion, and retention; (2) feasibility of screening processes; (3) utility of data collection and outcome measures; and (4) acceptability and suitability of the intervention and study procedures.

Our secondary objectives are to (1) evaluate participant opinions of the intervention as a basis for refinement of the definitive trial; (2) select a responsive and meaningful outcome measure; and (3) to inform the power and sample size calculations for the future full randomized controlled trial.

## Methods

### Trial Design

This study is a 3-armed randomized controlled trial design with a qualitative component. A total of 72 participants will be randomly allocated in a 1:1:1 ratio to a noisy galvanic vestibular stimulation group, sham group, or no intervention control group. Recruitment will close once 24 participants have been recruited into the noisy galvanic vestibular stimulation arm. Outcome assessment will occur at baseline (week 1), posttreatment (week 10) and at the 3-month follow-up posttreatment (Table 1).

**Table 1:** Standard Protocol Items-Recommendations for Interventional Trials tabulation of study enrollment, interventions, and assessments.

Study events		Study period						
		Enrollment	Postallocation					Closeout
			Week 1	Week 2-9	Week 10	Week 22		
**Eligibility screen**								
	STEADI^a^ questions	✓						
	Three-step command	✓						
	Demographics (age, sex, and ethnicity)	✓						
	Exclusion screen for nGVS^b^	✓						
	Informed consent	✓						
	Written informed consent		✓					
	Allocation	✓						
**Assessments**								
	Montreal Cognitive Assessment		✓					
	Activities-specific balance confidence		✓		✓	✓		
	Center of pressure		✓		✓	✓		
	Single and dual task gait		✓		✓	✓		
	Timed Up and Go		✓		✓	✓		
	Four-Stage Balance Test		✓		✓	✓		
	Functional Gait Assessment		✓		✓	✓		
	30-second chair stand test		✓		✓	✓		
	Triangle completion task		✓		✓	✓		
	4 Mountains Test		✓		✓	✓		
	Adverse event monitoring			✓				
	Intervention fidelity monitoring			✓				
	Semistructured interviews				✓			
**Intervention**								
	Exercise+nGVS			✓				
	Exercise+sham nGVS			✓				
	Exercise (control)						✓	

^a^STEADI: Stop elderly accidents, deaths, and injuries.

^b^nGVS: noisy galvanic vestibular stimulation.

Semistructured interviews posttreatment will investigate the acceptability of the research processes and the intervention from the perspectives of study participants and physiotherapists.

### Participant Recruitment and Eligibility Criteria

#### Recruitment

Older adults will be recruited from the community in a staged manner to determine the feasibility of recruitment methods. Suitable candidates will be approached via newspaper advertisements, local medical practices, newsletters targeting older adult community groups, retirement villages, social media, community notice boards, presentations to local groups, and professional networks. Data will be collected on the efficacy of each method in terms of response rate, proportion of respondents who were eligible, eligible respondents who subsequently consented to the study, and the number of participants who completed the study.

#### Screening

People interested in participating will contact a member of the research team via telephone or email. Initial screening and eligibility will be assessed by a trained research assistant via telephone and data entered into the REDCap database. Eligible participants who provide consent will be enrolled into the study. 

#### Inclusion Criteria

Participants are eligible for inclusion if they meet the following criteria: (1) aged ≥65 years or Maori or Pasifika aged ≥55 years, (2) independently living in the community, (3) considered at risk of falls based on the stop elderly accidents, deaths and injuries (STEADI) fall screening questions (Textbox 1) [27], and (4) able to stand independently for 5 minutes and ambulate for 10 m with or without a walking aid.

**Stop elderly accidents, deaths and injuries (STEADI) fall risk screening questions.**

**Screening questions (answering yes to any of these questions indicates a fall risk)**

- Have you fallen in the past year?
- Do you feel unsteady when standing or walking?
- Do you worry about falling?

#### Exclusion Criteria

Participants will be excluded if any of the following criteria are present: (1) diagnosis of a neurological condition, (2) pacemaker, (3) metal implants in the head or neck, (4) vertigo, (5) current diagnosis of migraine, and (6) cognitive impairment to the extent that they are unable to follow a 3-step command on the telephone.

#### Randomization

Participant group allocation will be by stratified random computer generation using a “minimization” algorithm in MinimPy (Msaghaei) to stratify groups by age, sex, and fall history in the past 6 months [28-30]. Allocation will be concealed in an opaque envelope identified only by the identification number of the participant and given to the participant after the initial assessment. Participants will be requested to open the envelope only after exiting the testing room. A contact number will be included in the envelope if participants wish to discuss their allocation.

#### Blinding

The noisy galvanic vestibular stimulation and sham group participants, assessors, therapists, and data analysts will be blinded to the group allocation. Measures will be taken to minimize the potential for the assessor and treating therapist to become unblinded. This will include avoiding discussions about the participants in front of the assessor and treating therapist. When participants are contacted to make appointments for the assessment, the team members involved will use this opportunity to remind participants not to reveal details of any treatment they have received to the assessor. A register of the unblinding events will be maintained.

#### Assessment

Outcome measures will be collected by a single investigator who will be blinded to the allocation and not involved in any aspect of the intervention.

#### Data Collection Methods

Once participants are recruited to the study and have given consent, they will attend three assessments at baseline, posttreatment, and at the 3-month follow-up (Table 1). Assessors will be trained in a protocol by a member of the research team who is familiar with the testing procedures. Assessments will be audited for quality and data completeness and checked for precision by the project manager once 10% of the data has been collected. A summary of the checks will be provided to the data monitoring committee. Any reports of adverse events will be reported and discussed at the monthly steering committee meeting.

### Outcomes

#### Participant Characteristics

The participant population will be described using demographic data, anthropometric data, including weight and height, the Montreal Cognitive Assessment, a brief cognitive screening tool [31], and the Activities Balance Confidence Scale, a structured questionnaire measuring an individual’s confidence performing specific activities [31].

#### Postural Sway Parameters

Postural sway will be calculated from center of pressure (COP) excursions on a force plate [4] (AMTI) in eyes open and eyes closed conditions. COP values of velocity, area, and root mean square during static standing have been shown to be precise, quickly administered assessments [32,33], with minimal ceiling and floor effects [34,35], and excellent sensitivity in the identification of older adult fallers [36-38]. In addition, we will investigate the relationship between the COP excursion and velocity and acceleration profiles of the head.

#### Gait and Mobility Performance–Based Measures

Performance-based measures of balance, gait, and leg muscle strength will indicate change in function and postural control. These tests are widely used in research for older adults with and without pathology, and all have norm-referenced values and robust clinimetric properties.

Gait will be measured using a 7 m long × 0.6 m–wide instrumented walkway, GAITrite, gold software version 3.2b, (CIR systems), in both single and dual task conditions [39]. Spatiotemporal parameters of interest include step velocity, length, width, double support time, and variability [40,41]. In addition, we will investigate the relationship between these spatiotemporal measures and the velocity and acceleration profiles of the head. A 9-camera motion capture system sampled at 200 Hz will record 3D kinematic data during the balance and gait measures. The 3D kinematic data will be exported to MATLAB (MathWorks, Inc) where a 6 degrees of freedom model will be constructed from shoulder and head marker position data. The model will consist of two triangles in 3D space, the first one corresponding to the shoulders and the second one for the head. The time series for the roll, pitch, and yaw angles of the head will be calculated with respect to the shoulders. Linear displacement across the mediolateral and anterior-posterior axes and the angular displacement about the vertical axis will be computed from these time series for a quantitative analysis of the head movement.

A range of mobility performance measures will be used. The Functional Gait Assessment assesses balance during walking under different conditions (speed change, head turn, pivot turn, obstacle clearance, and narrow base) [42,43]. The 30-second chair stand test measures functional leg strength. Participants will be asked to stand up from a chair and sit back down as often as they can within 30 seconds [44]. The Timed Up and Go test is a simple test used to assess the mobility of a person and requires both static and dynamic balance. It involves timing a person rising from a chair, walking 3 m, turning around, walking back to the chair, and sitting down. The Timed Up and Go test is considered a valid, reliable, and sensitive measure of mobility and balance in older adults [45,46]. For the Four-Stage Balance Test, participants are asked to stand in 4 progressively more challenging positions for 10 seconds with their eyes open and without using an assistive device. If the patient can hold a position for 10 seconds without moving their feet or needing support, they go on to the next position. Otherwise, the test is stopped. An older adult who cannot hold the tandem stance for 10 seconds is considered at increased risk of falling [47].

#### Spatial Memory

Spatial memory is strongly influenced by vestibular inputs [35,48] and will be assessed using the triangle completion task and the 4 Mountains Test. The triangle completion task combines vestibular and somatosensory inputs and interacts with cognitive and motor processes to perceive self-motion and generate a cognitive map of space [49-51]. The 4 Mountains Test uses a delayed match-to-sample paradigm on a computer-based image [52]. This is a highly sensitive test in older adults with mild cognitive impairment and can be easily applied in routine clinical practice [53].

####  Qualitative Assessment

A qualitative assessment in the form of a semistructured interview designed to investigate the acceptability of the treatment will occur at week 10 for selected noisy galvanic vestibular stimulation and sham participants. We aim to interview 5-6 participants in the noisy galvanic vestibular stimulation intervention group with a sample purposively selected to represent variation in intervention compliance and response; 2 sham participants will also be interviewed to explore the acceptability of the trial process. Physiotherapists delivering the intervention will be interviewed to explore the study processes, acceptability, and value of the intervention from their perspective. Semistructured interviews will be transcribed verbatim and uploaded to NVivo. We will conduct thematic analysis of the data using the 6-stage process of analysis (transcription, reading and familiarization, coding, searching for themes, reviewing themes, defining and naming themes, and finalizing the analysis) by Braun and Clarke [54]. Data will be initially analyzed at a semantic level to draw on the reflections of participants in relation to processes and practical aspects of the assessments and interventions. However, researchers will also identify latent themes, such as underlying ideas and assumptions, which are theorized to shape and inform semantic data.

### Intervention

The balance retraining program focuses on exercises and activities that stimulate the vestibular system and the integration of all sensory inputs for effective postural control (Table 2) [7,45,47]. The exercises are delivered in a group setting of up to 6 participants with difficulty in the exercises being individualized for each participant. The balance retraining program is delivered by an experienced and registered physiotherapist with 1 assistant for groups of 4 and above. Participants in the noisy galvanic vestibular stimulation group will receive 30 minutes of white noise galvanic vestibular stimulation during the exercise program; the sham group will receive sham stimulation during the exercise program, and the control group will receive no intervention [4,21]. The noisy galvanic vestibular stimulation generates a 0 mean random signal with equal intensities at different frequencies via electrodes applied over the mastoid process, which are attached to a noisy galvanic vestibular stimulation device (Galvanic Stimulator 0811, Soterix Medical). The intensity is set at 0.5 mA and is of imperceptible magnitude.

**Figure 1:**
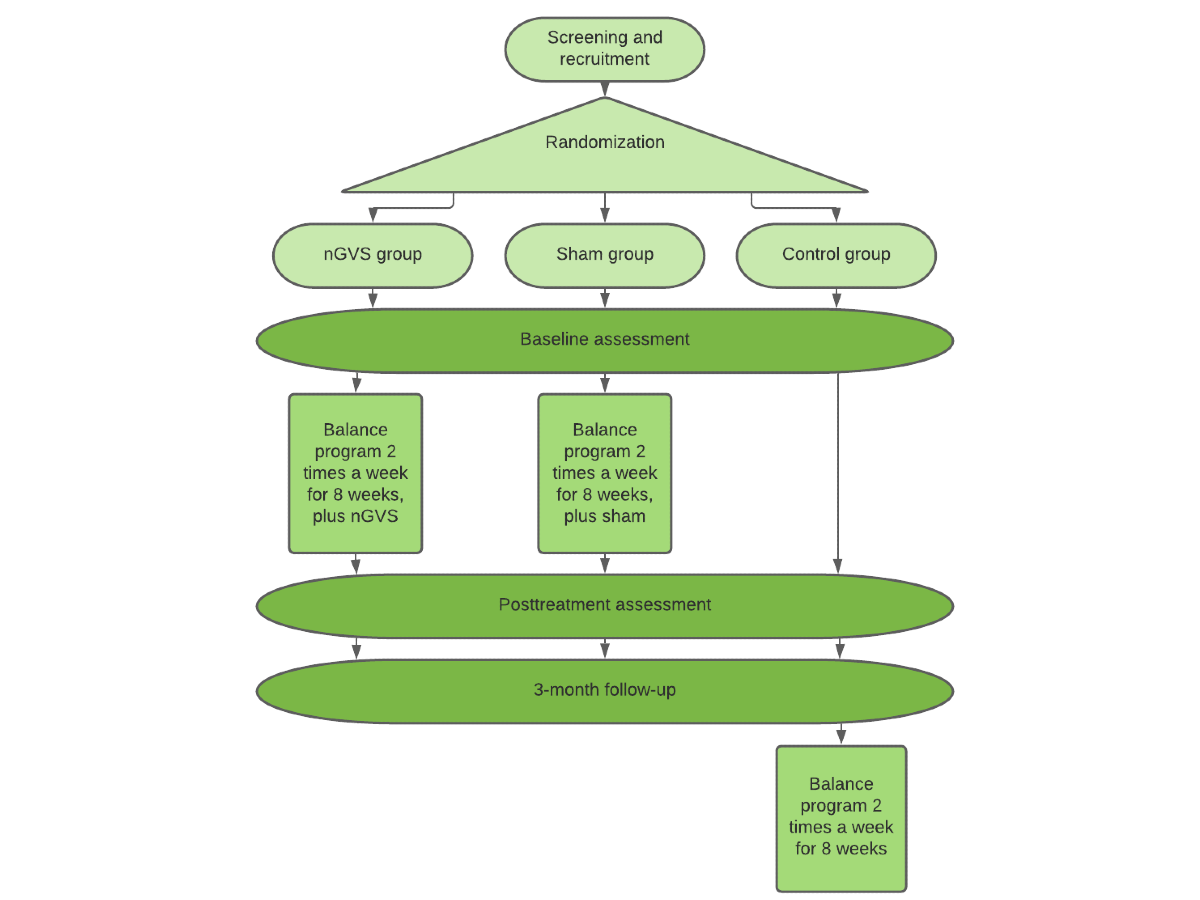
Participant flow through the study. nGVS: noisy galvanic vestibular stimulation.

The noisy galvanic vestibular stimulation and sham groups will commence the exercise program within 10 days of the initial assessment. The exercise group will be offered to the control group after the 3-month follow-up assessment at the end of their participation in the study (Figure 1).

**Table 2:** Summary of the multisensory balance program.

Category	Time (minutes)	Examples of exercises
Warmup	5	Multidirectional walking, turning, changing speed and size of movement
Vestibular	10	Standing, head turns with a target- fixed- gaze stimulating the vestibular ocular reflex. Standing on unstable surfaces eyes closed
Vision	5	Eyes open walking on narrow beam and standing on unstable surfaces
Somatosensory	5	Testing individuals’ limits of stability; multidirectional lean, standing on unstable surfaces
Mobility-center of gravity control	5	Walking a straight line, weaving and turning during gait, changing base of support and posture simultaneously while moving from low to high surfaces
Aerobic	5	Exercycle

### Data Management

Data will be entered directly into customized REDCap digital data collection forms; any paper forms will be uploaded into the REDCap data management system. REDCap access is password-protected and requires dual identification for entry. Forms will have internal checks for completeness and where appropriate individual criteria will have automatic range checks. All data will be deidentified on exportation from the database with analyses performed by a blinded member of the research team. Blinding will not be lifted until data collection and final follow-up assessment has been completed. Data will only be accessible to the research team. The results will be made available to participants on request and will be published in a peer-reviewed journal.

### Statistical Methods

The analysis will focus on univariate models, such as linear mixed models, but include measures of association. An examination of within-subject data will be carried out to measure changes in key outcomes. This will help refine participant selection, protocol, and suitability of outcome measures for the full study. Responsiveness metrics such as effect size and standardized response mean will be calculated for postural control outcomes to inform the selection of the primary outcome and power calculations for the intervention study.

Semistructured interviews will be transcribed verbatim and uploaded to NVivo. Data will undergo thematic analysis using the Braun and Clark six-stage process of analysis (transcription, reading and familiarization, coding, searching for themes, reviewing themes, defining and naming themes, and finalizing the analysis) [54]. Data will be initially analyzed at a semantic level to draw on the reflections of participants in relation to processes and practical aspects of the assessments and interventions. However, researchers will also aim to identify latent themes—the underlying ideas and assumptions that are theorized to shape and inform semantic data.

### Threats to Attainment of Study Goals

#### Overview

As a feasibility study investigating a novel treatment, there are a number of aspects of the study that could impact both the attainment of study goals and progression to a fully powered study. Possible threats identified by the study team are the inability to recruit, poor tolerance for noisy galvanic vestibular stimulation, technical issues with noisy galvanic vestibular stimulation, and adverse reactions to noisy galvanic vestibular stimulation. Although there have been very low rates of adverse effects with noisy galvanic vestibular stimulation, the participants in this study receive a larger cumulative dose of noisy galvanic vestibular stimulation compared with previous studies.

The trial will be deemed a success based on the following: (1) recruitment at a rate of 2 participants per week; (2) participants attending 75% of available exercise sessions over 8 weeks; (3) achieving 80% data completeness at each time point; (4) report of no serious adverse events related to the intervention; and (5) a subjective sense of perceived benefit, enjoyment, and engagement in the program present in participants who underwent qualitative assessment.

On the basis of previous research, we estimate that a sample of 72 would be sufficient to investigate feasibility and to determine the sensitivity of our outcome measures to inform sample size calculation for the full study.

#### Monitoring

The project manager will manage the day-to-day running of the study. Before study commencement, training sessions for clinical and research staff will be conducted. Fidelity will be ensured by the procedures outlined in Table 3. The data monitoring committee will consist of the research team, DT, SL, PFS, YW, and RM, who will meet monthly via Zoom. They will monitor the study to meet fidelity goals, adverse events, and unintended effects. Adverse events will be recorded on an incident form by the team member concerned, and the project manager and principal investigator will be informed by email or phone as appropriate. They will choose a course of action according to the study protocol and inform all relevant members of the research team. Compensation to those who are harmed during the trial is available to participants via the Accident Compensation Corporation, pending an eligible claim. It will be the responsibility of the principal investigator in discussion with the data monitoring committee to make the final decision to terminate the trial.

**Table 3:** Intervention fidelity.

Program component		Definition	Success criteria
**Staff training**			
	Training	Protocols are standardized and training involves didactic teaching, role playing and modeling	Protocol read and training modules completed by all treating physiotherapists
	Supervision	Frequency and duration of supervision are set out	Therapists are supervised in their initial treatment sessions and then have “spot checks” for compliance with the exercise program, progressions and documentation. All therapists have a contact number of the PI^a^ and project manager in the case of an adverse event or self-identified queries or supervision requirements
	Measurements	Establishing compliance with delivery of treatment	Treatments recorded on a customized, standardized form. Checked by the project manager to ensure data completeness
**Recruitment**			
	Methods	Staged approach to advertising	Data collected regarding methods of recruitment and number of people who responded via each method, response rate, number of responders sent a participant information sheet after initial contact
	Eligibility	Number of responders who were eligible and consented to the study. Reasons for ineligibility or deciding not to consent	Screening data recorded on a standardized form Average recruitment over the study of 2 participants per week
**Intervention delivery **			
	Intervention differences	Dose of exercise and noisy galvanic vestibular stimulation intervention	Expected dose and core components of the program as per the intervention protocol were recorded on the standardized program record
	Therapist competence	Experience and competence	Years of experience and areas of practice recorded for all treating therapists
	Monitoring drift	Ensure program delivered correctly throughout the program	Physiotherapists completed and returned a standardized record of all treatment visits. One visit per program between week 6 and 8 by a PI or project manager to ensure program continues to be delivered correctly
	Corrective feedback	Feedback procedures in place	Ongoing support and mentoring available if discrepancies noted during monitoring of treatment programs and documentation
**Intervention receipt**			
	Dose received	Data collected regarding the number of sessions attended for each participant If a participant does not attend, they are followed up with a phone call to assess any barriers to attendance Petrol vouchers will be given to defray travel expenses	Goal of 75% exercise sessions attended over 8 weeks
	Participant understanding	Qualitative interview	A subjective sense of perceived benefit, enjoyment, and engagement in the program present in participants who underwent qualitative assessment
	Participant adherence	Data collected regarding completion of follow-up data collection sessions. Participants may be contacted by phone, email and mail depending on their preference and every effort will be made to provide a mutually convenient time for the assessment Petrol vouchers will be given to defray travel expenses	Checks for data completeness, 95% of data present at each time point

^a^PI: principal investigator.

### Ethical Considerations

Ethics approval has been obtained from the Health and Disability Ethics Council New Zealand (20/STH/111), with the approval of the Auckland University of Technology Ethics Committee (20/310). The trial has been prospectively registered with the Australia New Zealand Clinical Trials Registry (ACTRN12620001172998) and has a Universal Trial Number of U1111-1241-2231.

Any amendments to the protocol will be done with approval from the Health and Disability Ethics Council, and the locality will also be informed (via the Auckland University of Technology Ethics Committee).

## Results

Recruitment for our pilot study began on November 26, 2020 the protocol version is 3.0 July 2, 2021. Initial recruitment was slow due to the COVID-19 pandemic, and some exercise groups were affected due to short periods of restrictions on gatherings affecting our region. However, despite these challenges, data collection and analysis will be completed by December 2022.

## Discussion

This study aims to deliver novel findings relevant to older adults at risk of falls. This study is the first step toward determining whether noisy galvanic vestibular stimulation can effectively and safely enhance balance and stability in a balance retraining program. Preliminary evidence suggests that the use of subsensory noisy galvanic vestibular stimulation to enhance vestibular signals to the brain also improves stability during quiet standing and gait [4,20,21,25,26]. This study will assess the feasibility of administering a balance retraining program augmented by noisy galvanic vestibular stimulation for older adults at risk for falls. The results of this study will inform the design of a future definitive randomized controlled trial.

Designing a trial to be conducted in a community setting presents challenges. Although community exercise programs are popular among older adults, older adults at risk of falling are a more vulnerable group who may have less access to the community or have anxiety about their stability and therefore be less represented in these groups. There has also been a public health push worldwide for older adults to *stay home and stay safe* during the ongoing COVID-19 outbreak. This has potentially led to physical barriers to community involvement, as well as mental and emotional barriers to commitments outside the home.

## Appendix

Multimedia Appendix 1 Peer review reports from the Health Research Council of New Zealand.

## Abbreviations

COP: center of pressure
STEADI: stopping elderly accidents, deaths and injuries
