# Supplementary material for: Noisy Galvanic Vestibular Stimulation Combined With a Multisensory Balance Program in Older Adults With Moderate to High Fall Risk: Protocol for a Feasibility Study for a Randomized Controlled Trial
Source: JMIR Res Protoc. 2021 Oct 5;10(10):e32085. doi: 10.2196/32085 (PMC8527374; doi:10.2196/32085)
Supplement: Multimedia Appendix 1 [file resprot_v10i10e32085_app1.pdf]

Health Research Council of New Zealand

3 May 2019

Professor Denise Taylor
Health & Rehabilitation Research Institute
Auckland University of Technology
Private Bag 92006
Victoria Street West
Auckland 1142

Tēnā koe Denise

Feasibility Study Application for Health Research Council Funding
HRC Reference: 19/632
Enhancing balance in older adults via noisy Galvanic Vestibular Stimulation

The Health Research Council of New Zealand (HRC) has completed the assessment of all Feasibility Study proposals for the 2019 funding round. I am pleased to advise that your proposal has been successful. The Council has offered funding to the level set out on the attached draft Third Schedule.

Please note that budgetary changes to your original application may necessitate a change in your research objectives. These changes, or other enquiries relating to the administrative aspects of your funding, should be directed to your Research Office. Once all changes have been agreed, contract documents will be sent to your Research Office for signing. A contract will not be formed until the HRC receives a completed "Staff Declaration - HRC Contracts form" (available from your research office) and copies of amended objectives and milestones (Fourth Schedule) and, any special conditions or requirements set out in the draft Third Schedule have been met. The *HRC Rules*, which form part of the contract, is available on the HRC website.

Some key conditions of the contract include best efforts to complete the proposed research, fulfilment of reporting requirements noting problems or delays as soon as they occur, changes or significant absences of key staff and significant changes to research objectives/ milestones. In your acceptance of this offer please indicate for our records whether an ethical approval is required for the planned research. Regular reporting aims to identify any issues or concerns as well as highlight positive outcomes of the research. Please let us know directly of any newsworthy impacts of our funding. Contract variations, such as time extensions, must be submitted to the HRC by your research office. All research reports can be now submitted on the HRC Gateway.

Your Research Office has been requested to return the draft Third and Fourth Schedules and signed Staff Declaration form by 17 May 2019. The Staff Declaration form should indicate time only staff and their FTE on the contract. Your Research Office is required to return the signed contract to the HRC by 7 June 2019. Unless your Research Office has received written authority from the HRC, your contract must commence no later than 1 July 2019. The funding may be withdrawn and returned to the HRC funding pool if this condition is not met.

Please note that the HRC will be making a media announcement about the outcome of this round in mid May 2019. Media activities initiated by your institution may follow the HRC's announcement but must not be before this announcement. This includes posting any result details on your websites. Please contact the HRC if you would like us to provide comment for your institution's media funding announcements as we would be happy to do so.

Some of the points raised during the assessment of your application are outlined in the enclosed Review Summary. If you wish to discuss the result of your application please address your enquiry, in the first instance, to your host institution and request that they write to Luke Garland, Manager Research Investment, at the HRC.

Note that all investigators receiving contract funding from the HRC must make themselves available, as reviewers or assessing committee members whenever possible. Please update your HRC Gateway profile to nominate yourself for HRC assessing committee membership.

I would like to add a personal note of congratulations on your success and I look forward to hearing of the progress and outcomes of your research.

Ngā mihi

Well done to you + your team Denise.

Professor Kathryn McPherson
Chief Executive

enc Review Summary
Draft Third Schedule
Draft Fourth Schedule

cc Auckland University of Technology Research Office

Feasibility Study Review Summary

**HRC Reference #**	19/632	**Applicant Surname**	Taylor
**Title of Research**	Enhancing balance in older adults via noisy Galvanic Vestibular Stimulation		
**Host**	Auckland University of Technology		

With regard to the criteria for assessing and scoring research proposals:

1. **What issues were considered by the Science Assessing Committee as important enough to influence the scoring of this proposal? (200-300 words)**

- The committee thought this was an interesting idea and offered real potential for enhancing balance.
- The committee felt there was a missed opportunity in terms of Māori engagement and suggested establishing relationships with Māori to enhance this opportunity.
- There were questions about how the investigators would decide which measures were the most sensitive and suggested that an *a priori* robust description be considered.
- There were questions about the generalisability of the trial. The investigators mentioned neurological conditions in the rationale, yet excluded this in the trial. This could affect generalisability and the committee suggested consideration be given to including these groups.
- The committee queried the acceptability of nGVS and noted that the team have limited expertise in qualitative methods (which are required to determine acceptability). The committee suggested the team consider including more expertise in this area.

2. **Other Comments**

- The committee wondered whether a $20 voucher was likely to reduce inequity in terms of access?

THIRD SCHEDULE SUMMARY - RESEARCH ACTIVITY DETAILS AND FUNDING

Research Provider: Auckland University of Technology

Contract Type: Feasibility Study

Contract Number: 19/632

First Named Investigator Professor Denise Taylor

Named Investigators: Professor Paul Smith, Dr Yiwen Zheng, Dr Sue Lord

DRAFT

Title: Enhancing balance in older adults via noisy Galvanic Vestibular Stimulation

Start Date: 1/08/2019 **Completion Date:** 31/07/2021 **Term:** 24

Organisations Sharing in Funding:

Reporting Dates: Annually on the anniversary of the grant plus 1 month

Budget Note: Incl. $55,893 funds for subcontract with University of Otago, to be released at contract commencement. Two months payment retained subject to satisfactory end of contract report.

Budget Outline (GST Exclusive) | **$**

**Total Salary:**	22,503.00
**Total W. Expenses:**	201,438.00
**Total Overheads:**	25,878.45
**Total Budget:**	249,819.45
**Administered by HRC:**	0.00
**Host Budget:**	249,819.45

Monthly Payment:

10,409.14

Payment Process:

monthly on the 20th day of the month

Investigators/Staff/Key Personnel

Professor Denise Taylor	0.05
Dr Sue Lord	0.03
Professor Paul Smith	0.05
Dr Yiwen Zheng	0.03
Recruiter ,	0.04
Assessor	0.15
Physiotherapist	0.07
Project Manager	0.11
Research Assistant	0.03
**Total FTE:**	**0.56**

Personnel marked * have a time commitment only

DRAFT

FOURTH SCHEDULE -
RESEARCH OBJECTIVES AND MILESTONES

Note that this page will form the basis of the contract for progress reports

#	Objectives
1	Determine recruitment capacity and sample characteristics
2	Complete data collection
3	Determine acceptability and suitability of the intervention and study procedures
4	Evaluate participant responses to the intervention
5	Dissemination

Contract Number:19/632 Date Printed: 29-Mar-2019

Year # for Completion of Milestones for each Objective		
**Year #**	**Milestones**	**Objective(s)**
1	Recruit participants for 3 groups in each arm	1
1	Determine data collection duration	2
1	Complete 5 interviews investigating participant burden in relation to data collection procedures.	2
1	Complete 5 participant interviews to determine the acceptability of the intervention and study procedures	3
1	Complete interviews withphysiotehrapists to determine intervention acceptability	3
2	Recruit participantsfor remaining 3 groups in each arm	1
2	Complete remaining participant interviews to ascertain participant burden with data collection procedures.	2
2	Determine the optimal COP measure to use in the Full Study	2
2	Finalise choice of outcome measures for Full Study	2
2	Evaluate retention and adherence rates	3
2	Explore the data to determine responses to the various outcomes measures	4
2	Complete remaining interviews to determine intervention acceptability	4
2	Provide summary feedback to participants	5
2	Submit the feasibility study for publication	5

Contract Number:19/632 Date Printed: 29-Mar-2019

DRAFT
